# Searching and synthesising ‘grey literature’ and ‘grey information’ in public health: critical reflections on three case studies

**DOI:** 10.1186/s13643-016-0337-y

**Published:** 2016-09-29

**Authors:** Jean Adams, Frances C. Hillier-Brown, Helen J. Moore, Amelia A. Lake, Vera Araujo-Soares, Martin White, Carolyn Summerbell

**Affiliations:** 1 Present address: Centre for Diet and Activity Research, MRC Epidemiology Unit, University of Cambridge, CB2 0QQ Cambridge, UK; 2 Institute of Health and Society, Newcastle University, NE2 4AA Newcastle, UK; 3 Fuse, The Centre for Translational Research in Public Health, Newcastle, UK; 4 School of Medicine, Pharmacy and Health, Durham University, TS17 3BA Stockton-on-Tees, UK; 5 Centre for Public Policy & Health, Durham University, TS17 6BH Stockton-on-Tees, UK

**Keywords:** Grey literature, Grey information, Systematic review, Evidence synthesis, Public health, Interventions

## Abstract

**Background:**

Grey literature includes a range of documents not controlled by commercial publishing organisations. This means that grey literature can be difficult to search and retrieve for evidence synthesis. Much knowledge and evidence in public health, and other fields, accumulates from innovation in practice. This knowledge may not even be of sufficient formality to meet the definition of grey literature. We term this knowledge ‘grey information’. Grey information may be even harder to search for and retrieve than grey literature.

**Methods:**

On three previous occasions, we have attempted to systematically search for and synthesise public health grey literature and information—both to summarise the extent and nature of particular classes of interventions and to synthesise results of evaluations. Here, we briefly describe these three ‘case studies’ but focus on our post hoc critical reflections on searching for and synthesising grey literature and information garnered from our experiences of these case studies. We believe these reflections will be useful to future researchers working in this area.

**Results:**

Issues discussed include search methods, searching efficiency, replicability of searches, data management, data extraction, assessing study ‘quality’, data synthesis, time and resources, and differentiating evidence synthesis from primary research.

**Conclusions:**

Information on applied public health research questions relating to the nature and range of public health interventions, as well as many evaluations of these interventions, may be predominantly, or only, held in grey literature and grey information. Evidence syntheses on these topics need, therefore, to embrace grey literature and information. Many typical systematic review methods for searching, appraising, managing, and synthesising the evidence base can be adapted for use with grey literature and information. Evidence synthesisers should carefully consider the opportunities and problems offered by including grey literature and information. Enhanced incentives for accurate recording and further methodological developments in retrieval will facilitate future syntheses of grey literature and information.

## Background

Public health researchers may want to include ‘grey literature’ in evidence syntheses for at least three reasons. Firstly, including grey literature can reduce the impact of publication bias as studies with null findings are less likely to be published in peer-reviewed journals [1]. Secondly, grey literature can provide useful contextual information on how, why, and in whom complex public health interventions are effective [2–6]. Finally, syntheses of grey literature can help applied researchers and practitioners understand what interventions exist for a particular problem, the full range of evaluations (if any) that have been conducted, and where further intervention development and evaluation is needed.

Numerous definitions of grey literature exist. These tend to focus on the fact that it is not controlled by commercial organisations, making it difficult to search for and retrieve [7–9]. One common definition restricts grey literature to literature ‘protected by intellectual property rights, of sufficient quality to be collected and preserved by library holdings or institutional repositories’ [10]. Other definitions are more inclusive and propose that, given the growth of new forms of media, grey literature should not be restricted to written ‘literature’ [4].

Much knowledge and evidence in applied settings, such as public health practice, accumulates from innovation in practice [7]. This may include the rationale for why new approaches were tried; what changes, if any, were made to previous approaches and why; what was done and how; and what happened. In some cases, this may be accompanied by more formal process evaluation and, most rarely, outcome evaluation (Hillier-Brown F, Summerbell C, Moore H, Wrieden W, Abraham C, Adams J, Adamson A, Araujo-Soares V, White M, Lake A. A description of interventions promoting healthier ready-to-eat meals (to eat in, to take away, or to be delivered) sold by specific food outlets in England: a systematic mapping and evidence synthesis. BMC public health 2015, unpublished). Interventions and evaluations that were primarily conducted as part of, or to inform, practice may be particularly unlikely to be described in peer-reviewed publications or even formally documented in reports available to others in electronic or hard copy. Information on these activities may, instead, be stored in more private or informal spaces such as meeting notes, emails, or even just in people’s memories. This information is likely to be of insufficient formality to meet the definition of grey literature. We term this ‘grey information’.

The phrase ‘grey information’ has been used previously to extend the concept of grey literature to a wider range of sources [11], but it is not widely used and we are not aware of a previously stated clear definition. The term ‘grey data’ [12] has also been used specifically to describe user-generated web content—something that we feel is more formal and public than ‘grey information’ but less formal than ‘grey literature’. Table 1 describes defining aspects and examples of the three terms: grey literature, grey data, and grey information.

**Table 1:** Defining aspects and examples of ‘grey literature’, ‘grey data’, and ‘grey information’

Term	Defining aspect	Examples
Grey literature [7–9]	Not controlled by commercial publishing organisations	Internal reports, Working papers, Newsletters
Grey data [12]	User-generated, web-based	Tweets, Blogs, Facebook status updates
Grey information	Informally published or not published at all	Meeting notes, Emails, Personal memories

Systematically identifying grey literature and grey data is not a straightforward task [5, 7–9, 12, 13]. Systematically identifying ‘grey information’ is likely to be even more challenging. A number of case studies have been published describing procedures for searching and retrieving ‘grey literature’ in public health contexts [13, 14]. These tend to adopt relatively similar approaches including searching databases of peer-reviewed and grey literature; conducting structured searches of relevant websites and search engines; and contacting relevant experts [5, 8, 9, 13].

On three occasions, various authors of this article have attempted to systematically search for and synthesise public health grey literature and information. Here, we briefly describe our experiences of these three case studies and then critically reflect on these. ‘Critical reflection’ is a concept most often associated with adult learning and professional development. Although poorly and diversely defined, critical reflection is generally associated with post hoc examination of experiences in an attempt to improve future practice [15, 16]. Our aim was to provide insights on searching for and synthesising grey literature and information that may be useful for future researchers.

## Methods

The aims, methods, results, and conclusions of our three case studies are summarised in Table 2.

**Table 2:** Summary of aims, methods, and results of three case studies of searching for and synthesising grey literature and grey information

	Review 1, 2006 [17]	Review 2, 2011 [20]	Review 3, 2016 (Hillier-Brown F, Summerbell C, Moore H, Wrieden W, Abraham C, Adams J, Adamson A, Araujo-Soares V, White M, Lake A. A description of interventions promoting healthier ready-to-eat meals (to eat in, to take away, or to be delivered) sold by specific food outlets in England: a systematic mapping and evidence synthesis. BMC public health 2015, unpublished)
Aims	‘To answer the question: what are the health, social and financial impacts of welfare rights advice delivered in healthcare settings?’	‘Identify the range of existing adult cooking skills interventions that are presently implemented in England which meet key criteria…Make a judgement on the suitability of each identified intervention for rigorous outcome evaluation.’	‘To systematically identify interventions to promote healthier ready-to-eat meals sold by specific food outlets in England. To describe the type of interventions, and summarise information on their content and delivery. To summarise information from [any] evaluations.’
Inclusion criteria	Evaluations of welfare right advice in a healthcare setting in terms of health, social, or financial outcomes. No exclusions based on: Outcomes Study design Study population Place of publication Language of publication	Interventions that meet all the criteria: Aim to develop basic kitchen and cooking skills Target adults aged 16 years or over Target non-professional cooks Use a written curriculum Involve interaction between tutor and participant Involve more than one session Run on a not-for-profit basis	Interventions that meet all the criteria: In specific food outlets Openly accessible to the general public Selling ready-to-eat meals and beverages as their main business for profit No exclusions based on: place of publication/reporting of information Methodological quality
Search methods	Searches of: Databases of peer-reviewed and grey literature relevant journals an internet search engine relevant funder and third sector websites References and citations of included studies publications of authors of included studies targeted requests sent via email to those with publications in the field General requests: Sent to relevant email distributions lists posted on online bulletin boards published in ‘trade press’	Searches of: An internet search engine Relevant funder and third sector websites Targeted requests sent via email to: All Primary Care Trusts (PCTs) in England All local authorities (LA) in England All regional obesity leads in England Regional voluntary sector network organisations	Searches of: Databases of peer-reviewed and grey literature research and trial databases an internet search engine relevant funder and third sector websites media database Targeted requests sent via email to: All local authorities in England Those with publications in the field General requests: Sent to relevant professionals orgs via Twitter Sent to relevant email distributions lists Posted on online bulletin boards Published in ‘trade press’
Type of literature and information included	Peer-reviewed literature Grey literature	Grey literature Grey information	Peer-reviewed literature Grey literature Grey information
Synthesis method	Narrative, with quantitative synthesis of mean financial benefit per client	Narrative, with ‘theory mapping’ of interventions to identify the key behaviour change theories used	Narrative synthesis
Studies/interventions included (*n*)	55	14	102 (30 of which included an evaluation)
Conclusions	‘Welfare rights advice services can go some way to resolving under claiming. However, there is currently little evidence of adequate robustness and quality to indicate that such services lead to health improvements.’	‘We recommend that an outcome evaluation, involving a randomised controlled trial (RCT), a process, and an economic evaluation, is conducted…preceded by feasibility work.’ ‘*Jamie’s Ministry of Food* is the only single intervention identified that could fulfil the sample size requirements. However…this intervention may not make best use of behaviour change theory. A number of smaller interventions make good use of theory [but] would [not] fulfil the sample size requirements.’ ‘We recommend either or both of: *Jamie’s Ministry of Food* is approached to discuss their willingness to develop their programme, with a view to taking part in an RCT. Or, a number of existing local interventions, which make good use of theory, are approached to discuss if their programmes could be harmonised, with a view to taking part in an RCT.’	‘The best available evidence suggests that food outlet proprietors are generally positive about implementing these interventions, particularly when they are cost neutral and use a ‘health-by-stealth’ approach. Little robust evidence is available on the effectiveness of these approaches and further research is needed to generate this evidence. Opportunities for working upstream with suppliers, and in co-participation with consumers, when developing interventions should be explored.’

### Review 1: the health, social, and financial impacts of welfare right advice delivered in healthcare settings [17]

Our first review included grey literature alongside peer-reviewed literature in a systematic review of the health, social, and financial impacts of welfare rights advice delivered in healthcare settings [17]. In part, this systematic review was conducted in preparation for an application for funding for a randomised controlled trial of the impacts of welfare rights advice on health in older people [18, 19]. Thus, we were interested both in the extent and findings of other research. We conducted a quantitative synthesis of the average financial impacts of welfare rights advice and a narrative synthesis of other quantitative and qualitative findings.

As expected, less than half of the evaluations of welfare right advice included in the review were published in peer-reviewed journals. The remainder were published in reports published by delivery organisations, universities, other research organisations, and service and research funders.

### Review 2: adult cooking skill interventions in England [20]

Our second attempt to review grey literature explored the nature, content, and range, but not effects, of interventions seeking to enhance adult cooking skills delivered in England [20]. Our intention was to identify the most sustainable and theoretically promising of these to take forward for more formal evaluation. Similar to other reviews [21], our synthesis focused on categorising interventions according to delivery setting and training model and summarising the training delivered, throughput, setup and running costs, funding, and behaviour change techniques used [22].

This review focused entirely on grey literature and information and did not include any searching for peer-reviewed literature. A scoping review of peer-reviewed outcome evaluations of adult cooking skill interventions was conducted in parallel [23].

### Review 3: interventions promoting healthier ready-to-eat meals (to eat in, to take away, or to be delivered) sold by specific food outlets in England (Hillier-Brown F, Summerbell C, Moore H, Wrieden W, Abraham C, Adams J, Adamson A, Araujo-Soares V, White M, Lake A. A description of interventions promoting healthier ready-to-eat meals (to eat in, to take away, or to be delivered) sold by specific food outlets in England: a systematic mapping and evidence synthesis. BMC public health 2015, unpublished)

Finally, we recently completed a review of interventions aiming to promote healthier ready-to-eat meals (to eat in, to take away, or to be delivered) sold by food outlets in England (Hillier-Brown F, Summerbell C, Moore H, Wrieden W, Abraham C, Adams J, Adamson A, Araujo-Soares V, White M, Lake A. A description of interventions promoting healthier ready-to-eat meals (to eat in, to take away, or to be delivered) sold by specific food outlets in England: a systematic mapping and evidence synthesis. BMC public health 2015, unpublished). This explored the nature and range of interventions implemented and summarised evaluation findings. We used a narrative approach to evidence synthesis, characterising interventions, identifying issues of design and delivery, and summarising evaluation findings on process, acceptability, cost, and impact. Our intention in this case was to use the findings to inform development and evaluation of new interventions based on the most promising features of previous ones.

Whilst this review did include searches of peer-reviewed literature, these only identified one included study—although two other relevant peer-reviewed papers were identified using other methods. As in review 2, a linked review of peer-reviewed evidence was conducted in parallel [24].

## Results and discussion

Whilst there is much useful guidance available on evidence synthesis in general [25–27], and searching for and synthesising grey literature in particular [5, 8, 9, 13, 28–30], one size rarely fits all. Throughout, and in common with the best research practice, our methods were guided by our aims.

### Searching

In evidence syntheses, the sensitivity, specificity, and type of information retrieved by searches is highly dependent on the search strategy used. As described above and in Table 2, we used a variety of different methods to search for information across all three reviews. We reflect on some of the issues raised below and summarise some of our conclusions in Table 3.

**Table 3:** Characteristics of different approaches to searching for grey literature and grey information

Search method	Specific to grey literature?	Likely to find grey literature?	Specific to grey information?	Likely to find grey information?	Likely to be replicable?	Results likely to be up to date?	Easy for recipients to share?	Easy for recipients to ignore?
Searches of								
Databases of peer-reviewed literature	No	No	No	No	Yes	Yes	NA	NA
Databases of grey literature	Yes	Yes	No	No	Yes	Yes	NA	NA
Databases of media reporting	No	Yes	No	No	Yes	Yes	NA	NA
Relevant peer-reviewed journals	No	No	No	No	Yes	Yes	NA	NA
Internet search engines	No	Yes	No	No	Possibly	Yes	NA	NA
Reference and citations of included studies	No	No	No	No	Yes	Yes	NA	NA
Other publications of authors of included studies	No	No	No	No	Yes	Yes	NA	NA
Relevant funder and third sector websites	No	Yes	No	No	Possibly	Possibly	NA	NA
General requests for information sent to email lists, online boads, published in ‘professional press’ and distributed via Twitter	No	Yes	No	Yes	Possibly	Yes	Yes	Yes
Targeted requests sent via email to named contacts	No	Yes	No	Yes	Possibly	Yes	Yes	Possibly

*NA* not applicable

#### Search methods

In all three cases, and as recommended by others [5, 8, 9, 13, 28–30], we used a wide variety of methods to search for relevant grey literature and information. Across our three examples, we searched trial databases (e.g. www.isrctn.com), grey literature databases (e.g. www.opengrey.eu), websites of relevant organisations (e.g. charities with an interest in social inclusion in review 1), and a popular internet search engine (i.e. www.google.co.uk).

We also contacted those working in the areas we were interested in. We sent both personalised requests to key informants, as well as more generic requests to professional organisations and groups, using a variety of methods. In reviews 1 and 3, researchers working in relevant fields were contacted via email and requests for information were sent to relevant email lists, posted on online bulletin boards, and published in the ‘professional press’ (e.g. newsletters of professional organisations). In reviews 2 and 3, we also attempted to contact relevant individuals working in all local public health departments in England. In review 3, we incorporated social media into our search strategy.

Review 1 was conducted in 2005 when social media and social networks were less well established than they are now. To target large networks of professionals in this case, we published requests for help in the ‘professional press’. By the time review 3 was conducted, in 2014, social media had become an important space for professional networking. We posted numerous tweets requesting relevant information and asking those who saw them to repost (i.e. ‘retweet’) them to their own networks—in order to increase the potential number of people who saw these requests. Many of these tweets tagged (i.e. ‘@mentioned’) relevant professional organisations. We are not aware of previous reviewers using social media to identify grey (or peer-reviewed) literature or information.

This transition in methods from reviews 1–3 over just less than a decade reflects how information storage and sharing has changed over this time in the UK. At the same time, information storage and sharing patterns may vary internationally. Search methods need to adapt to local and international trends in information systems, and researchers should be flexible to this.

#### Searching efficiency

As with ‘typical’ systematic reviews [31, 32], our searching sacrificed specificity for sensitivity. Searches yielded many results that did not meet our inclusion criteria. The resource and scientific implications of the trade-off between search specificity and sensitivity have been widely discussed in the systematic review literature [33, 34].

Previous case studies have described very different ‘hit’ rates associated with different grey literature search strategies. In a review of interventions to promote walking and cycling, requests for help emailed to key informants added little to database searching [35]. Whereas, in a review of behaviour change interventions published only in grey literature, 70 % of items included in the final synthesis came from key informants [5]. Similarly, we found that different methods for locating information were differentially effective across our three reviews. In review 1, generic requests sent to email lists and published in the professional press were particularly useful. On a number of occasions, these requests were passed through a number of people before someone responded with relevant information—further adding to the time taken to conduct searching that is discussed below. Perhaps, similarly, in review 3, Twitter requests were particularly valuable. These were widely retweeted, vastly increasing the pool of potential viewers, but this appeared to be a much quicker process than cascading of email requests and requests in the professional press.

The efficiency of different search methods are, at least partly, dependent on the quality of the search strategy used. Simple comparisons, such as those described above, are not necessarily fair. Nor is it clear if the differences in efficiency are predictable. If the efficiency of different approaches to searching could be predicted in advance, this could help reviewers to focus their resources.

Our resources were most limited in review 2, and it became evident early in searching that we would not be able to complete a comprehensive search for all adult cooking skill interventions in England. We made a pragmatic decision to focus instead on identifying intervention types—based on delivery context and training model. As others have done [13], we borrowed the concept of ‘data saturation’ from qualitative research and stopped searching when we felt we were not identifying any new intervention types. We felt that sacrificing sensitivity in this case did not compromise our ability to meet our aims.

#### Using others to target searching

In reviews 2 and 3, we attempted to ask all local public health departments in England what relevant projects they were aware of. We are not aware of any peer-reviewed publications which report the efforts of other evidence synthesisers, or indeed primary researchers, who have attempted to systematically contact all local public health departments across one country in this way. That said, we recognise that the gathering of data on the activity and type of public health interventions conducted at various geographic levels is a relatively common activity. To facilitate this, we identified named individuals and contact email addresses for those with relevant roles using internet searches and telephone calls. This was a time-consuming task in itself. The requests for information we sent specifically asked recipients to pass our enquiries onto those they felt were best placed to respond. As with other email requests (see above), there were examples where messages had been passed through a number of individuals before someone responded.

#### Replicability of searches

Whilst in all cases, we had clear plans describing what we felt were comprehensive, systematic, and replicable approaches to information searching, it is hard to claim that these led to replicable results. Certainly, it would be possible for future investigators to replicate our search methods, but it is unlikely that these would lead to the same results on replication, as would be expected when using electronic databases. On two different occasions, different people would be likely to see calls for information on social networks or in the professional press. Even if the same people did see requests for help on different occasions, many other contextual factors may influence how likely they were to respond or pass them onto those most likely to be able to respond.

As time passes and grey literature and information becomes lost or forgotten, potential respondents’ ability to provide usable information may also decline. Whilst contacting both those currently and previously in posts as key informants may, theoretically, reduce this problem, it may not be practically possible. Others have highlighted the problem of replicability in relation to internet searching, particularly using search engines such as www.google.com which returns results based on, amongst other things, recent popularity [8, 9].

The conclusion that searching for grey literature and information can be systematic, but not necessarily replicable, reinforces the importance of using many overlapping searching approaches. This maximises the chances that any particular piece of relevant information will be found.

#### Developing the ‘best’ search methods

Whilst our search methods were similar to, and built on, those described by others as well as on ‘best practice’ guidance [5, 8, 9, 13, 28–30], it is difficult to be sure what the ‘best’ search methods for retrieving grey literature and information are. Whilst it is possible to validate search approaches in peer-reviewed literature against a ‘gold standard’ of hand searching [31, 32], no similar gold standard exists for grey literature and information: there is no definitive repository against which other search methods can be compared. This makes it difficult to ever be sure that all relevant information has been found or validate new search methods.

### Data management

In all three reviews, we found data management to be challenging. Technology now allows fairly straightforward integration of academic databases and reference management software—both of which facilitate information organisation and record keeping. Such workflows are not well developed for grey literature and information. Developing clear filing and recording systems, using simple spreadsheets, helped us to keep track of where and how information had been identified.

However, we found it harder to capture other aspects of our searches. For example, whilst tools like NCapture allow social media content to be imported in NVivo for qualitative analysis, they do not necessarily provide a useful facility for capturing how many people (and who) ‘retweeted’ a particular tweet. It is even harder to capture when requests for information are circulated using more private methods such as email. For these reasons, we are not able to provide accurate estimates of how many people saw our requests for information.

### Data extraction

In all three reviews, we developed and used data extraction forms to record information. In review 1, we adopted a similar approach to data extraction used in many ‘typical’ systematic reviews—if information was not provided in the written report we obtained, we assumed this information was missing. However, systematic review guidance encourages reviewers to attempt to minimise missing data by contacting authors of original papers [25]. We adopted an approach much more similar to this in reviews 2 and 3. In fact, many data extraction forms in these reviews were completed during telephone calls or following email conversations with key informants. To maximise accuracy, in review 3, we asked informants to check electronic versions of completed data extraction forms. As there is often little or no documentary evidence to refer back to, ensuring data extraction forms are as accurate and complete as possible is particularly important in reviews of grey literature and information. This reflects and reinforces the fact that much information on interventions in public health practice is not well documented and can be ‘temporary’: once the relevant individuals move to new posts, and interventions recede into the past, individual and institutional memories are likely to fade. This further contributes to the limited replicability of this sort of grey information searching.

Despite the efforts we made in reviews 2 and 3 to speak with those directly responsible for intervention design and delivery, we were often not able to obtain the information we intended to capture. For example, of 102 interventions identified in review 3, we were not able to obtain any information beyond a programme name in 27 cases. In most, if not all, cases, our failure to obtain information appeared to be because such information was not documented or easily obtainable. For example, many of the costs of public health interventions in everyday practice are unclear even to those responsible for them. Whilst the cost of additional staff may be explicit, costs for office space for those staff might be absorbed by organisations and so be much more implicit.

The problem of limited data availability is common to all types of evidence synthesis, but others have noted it as a particular problem when synthesising grey literature [7, 13]. When attempting to synthesise the extent of public health practice, it may be important to be aware of the types of information that are and are not important to practitioners and easy for them to record and hence are likely to be documented. For example, service throughput appears to be much more likely to be documented than outcomes of interventions (Hillier-Brown F, Summerbell C, Moore H, Wrieden W, Abraham C, Adams J, Adamson A, Araujo-Soares V, White M, Lake A. A description of interventions promoting healthier ready-to-eat meals (to eat in, to take away, or to be delivered) sold by specific food outlets in England: a systematic mapping and evidence synthesis. BMC public health 2015, unpublished) [17].

### Risk of bias and value of information

The risk of bias of any piece of information is dependent, in part, on the question it is being used to answer. In review 2, and part of review 3, our aims were to describe the nature and range of particular classes of interventions. The risk of bias of individual pieces of grey literature and information in this case is likely to be low—there is little reason why such information would be misrecorded. In contrast, in review 1 and part of review 3, we aimed to synthesise evaluation findings. The risk of bias of grey literature and information in this case may be likely to be higher. Indeed, in reviews 1 and 3, we described some aspects of evaluation methods relating to risk of bias. In both cases, we concluded that the majority of studies were methodologically weak and at high risk of bias.

Evaluations found in grey literature and information may be at high risk of bias for a number of reasons. In public health practice, evaluations are often conducted by the same practitioners who developed and delivered an intervention. This results in an inherent conflict of interest which may increase risk of bias. In public health practice, resources for evaluation are often limited, limiting the scope of what is possible [36]. Furthermore, the interest of funders and practitioners is often on throughput rather than outcomes [37], limiting the scope of what is necessary. Whilst many evaluations included in our reviews were at high risk of bias in terms of conclusions about effects on outcomes, they may well have been fit for the purpose for which they were conducted.

Methods for assessing risk of bias in controlled trials are well established [38, 39], and tools for other types of study are becoming available [40–42]. However, these approaches may be too narrow in perspective for grey literature and information. Realist synthesis takes a researcher-driven ‘value of information’ approach to assessing studies, rather than the more familiar protocol-driven risk of bias approach used in ‘typical’ systematic reviews. In the value of information paradigm, individual studies are included if the information they provide is considered relevant and rigorous enough to help contribute to answering the research question [6, 43, 44]. Whilst this requires researchers to make judgements about what is ‘relevant and rigorous enough’, it may result in inclusion of more potentially useful grey literature and information than stricter approaches which exclude studies based on risk of bias assessments.

### Data synthesis

Many approaches to data synthesis in the context of systematic reviewing and evidence synthesis have now been described, and these are not limited to quantitative meta-analysis [25, 45]. Although we performed a quantitative synthesis in review 1, this focused on the economic benefits of welfare rights advice to recipients (which could be summarised in £/week). We were not able to summarise health and social implications so simply and used narrative syntheses for these.

In review 3, in an attempt to capture all the data available to us, we adopted a three tiered approach to synthesis. Firstly, we listed all relevant interventions that we found (*n* = 102; tier 1). Secondly, for those interventions for which we had further information on content and delivery, we summarised this using a standard template (*n* = 75; tier 2). Finally, we summarised the results of any evaluations of included interventions (*n* = 30; tier 3). Interventions in each tier were nested within each other such that all interventions were included in the tier 1 synthesis, but only a subset of these were included in tier 2, and only a subset of those in tier 2 were included in the tier 3.

These differences in synthesis approach reflect both the contrasting aims of different reviews and how flexible and responsive researchers should be to the realities of data availability within grey literature and grey information.

### Time and resources

Systematic reviews can be time and resource intensive. In ‘typical’ systematic reviews, preliminary scoping reviews can help reviewers estimate the size of a full review and resources required [46]. ‘Rapid reviews’ of peer-reviewed literature offer the hope and potential for conducting much quicker evidence syntheses that arrive at the same conclusions as full reviews [47–49]. Unfortunately, there is no clear equivalent of scoping or rapid reviews in relation to grey literature and information. As others have noted, searching for less formally archived information is, almost by nature, time-consuming and inefficient [5, 8, 50].

Encouraging public health practitioners to deposit intervention documents and information in online repositories (e.g. www.ncdlinks.org) could enable more efficient information retrieval on current and recent practice [7]. But the utility of such databases relies heavily on their coverage, and previous attempts to ensure high coverage have been varying in their success [51]. With few obvious current incentives for busy practitioners to deposit information in these repositories, it is not necessarily clear how they could be made more useful. Further attention could be given to developing such incentives. In addition, developing better searching and retrieval methods should also facilitate syntheses of grey literature and information, particularly using more sophisticated methods for internet searching such as text analytics or data mining [7, 52]. However, if grey information is not recorded in a searchable way (e.g. is retained only on private networks or in memory), this is also only a partial solution. Action is required to improve both information deposition and information retrieval.

### Differentiating evidence synthesis from primary research

Although we approached and considered all three of our case studies as evidence syntheses, they could be considered as verging on primary research. This is particularly the case for reviews 2 and 3 where we made attempts to contact all local authorities in England and collect unpublished information via telephone or email interviews with key informants. Contacting authors is encouraged in ‘typical’ systematic reviews, particularly to collect information that may be incompletely recorded in published outputs [25]. This type of contact is not routinely considered primary research, as the contact is limited to clarifying or augmenting existing published information—rather than collecting entirely new data. However, in many cases in reviews 2 and 3, no published information was available to clarify or augment meaning that these reviews could, perhaps, be considered as collecting new data.

This grey area between evidence synthesis and primary research is particularly important in terms of research ethics. In general, research ethics committee review is not required for evidence synthesis projects because they do not involve research participants [53]. In line with this, we did not obtain research ethics committee review for any of the case studies described. It is not clear at what point ‘key informants’ become ‘research participants’ and hence when the type of evidence synthesis we conducted in reviews 2 and 3 becomes primary research that does require research ethics committee review. Further consideration, and clarification, of this issue by research ethics organisations would be helpful. In the meantime, and as has been previously proposed, it may be judicious for researchers proposing to conduct this type of work to at least discuss it informally with their local research ethics committee before proceeding [54].

## Conclusion

We propose the term ‘grey information’ to capture a wide range of documented and undocumented information that may be excluded by common definitions of ‘grey literature’. Information on applied public health research questions relating to the nature and range of public health interventions, and many evaluations of these interventions, may be predominantly, or only, held in grey literature and grey information. Evidence syntheses on these topics need, therefore, to embrace grey literature and information. Many standard systematic review methods for searching, appraising, managing, and synthesising the evidence base can be adapted for use with grey literature and information. Evidence synthesisers should carefully consider the opportunities and problems offered by including grey literature and information. Further action to improve both information deposition and retrieval would facilitate more efficient and complete syntheses of grey literature and information.
